# Correlation between serum inflammatory factors and cognitive function in patients with high-altitude polycythemia: A case–control study

**DOI:** 10.1097/MD.0000000000037983

**Published:** 2024-04-26

**Authors:** Yinglan Li, Jiabing Wang, Xiuxin Zhang, Qiong Ye, Yuan Yang, Xiaoshan Cui, Jinhua Feng, Jimei Li

**Affiliations:** a General Department, Qinghai Provincial People’s Hospital, Xining, China.

**Keywords:** cognitive function, high-altitude polycythemia (HAPC), inflammatory factors

## Abstract

The purpose of this study is to investigate the serum inflammatory factors in patients with high-altitude polycythemia (HAPC) and their correlation with cognitive function. The subjects were recruited and placed into a HAPC group and control group. Serum samples were collected, and inflammatory factors (interleukin-1beta [IL-1β], monocyte chemoattractant protein-1 [MCP-1], and tumor necrosis factor-alpha [TNF-α]) were measured using ELISA kits. The mini-mental State Examination (MMSE) was used to assess cognitive function. According to the MMSE scores, HAPC group was further divided into normal cognitive function group (HNCF) and cognitive dysfunction group (HCDF). In comparison with the control group, the MMSE scores in the HAPC group were significantly low (*P* < .05), whereas the serum levels of IL-1β, MCP-1, and TNF-α were significantly high (*P* < .01). Among the HAPC group (n = 60), 21 belonged to the HCDF and 39 belonged to the HNCF. Compared with the HNCF, the IL-1β, MCP-1, and TNF-α in the HCDF were significantly increased (*P* < .01). The Pearson correlation analysis showed that inflammatory factors were positively correlated with hemoglobin, and negatively correlated with MMSE. Serum inflammatory cytokines IL-1, MCP-1, and TNF-α were increased in HAPC, and HAPC exhibited cognitive dysfunction. Considering chronic hypoxia environment influences the change of the red blood cell metabolic and inflammatory factor, red blood cells and inflammatory factor in plateau is likely to be affected by patients with vascular lesions, increase cognitive impairment.

## 1. Introduction

High-altitude polycythemia (HAPC) is dependent on factors, such as altitude, occupation, sex, age, and lifestyle, and predominantly occurs in native inhabitants or long-term residents living at altitudes above 2500 m.^[1]^ Moreover, the pathogenesis of HAPC is complex.^[2]^ Studies have suggested that high-altitude hypoxia induces the abnormal expression of hypoxia-induced factor, which promotes inflammatory responses, hyperactivity of the hematopoietic system, and further promotes the occurrence of HAPC.^[2,3]^ It has also been found that HAPC is closely related to hematopoietic factors and inflammatory factors.^[3,4]^ HAPC can cause blood vessel damage, microvascular lesions, cerebral arteriosclerosis, cerebral perfusion, cerebral metabolism, and inflammatory responses in the brain promoting the release of inflammatory cytokines, peripheral blood cells, and peripheral blood of inflammatory cytokines, also changing the permeability of the blood–brain barrier and promoting inflammation factors and white blood cells into the brain, with decreased cognitive function.^[5–7]^ At present, there are few reports on the correlation between inflammatory factors and cognitive impairment in patients with polycythemia in altitude hypoxia environments worldwide.

To this end, this study aims to investigate the correlation between inflammatory cytokines and erythrocytosis in patients with polycythemia to identify the other factors that induce the onset of HAPC and to understand the network regulation effect among cytokines. In addition, the correlation between patients with polycythemia and cognitive function was studied to provide new insights into the clinical prevention and treatment of polycythemia in the plateau region.

## 2. Materials and methods

### 2.1. General information

During March to December 2016, 60 patients with polycythemia and 60 healthy individuals from Maduo County, Qinghai Province, China at elevations of 4300 m were selected as the subjects. Patients with polycythemia were further divided into 2 subgroups based on the Mini-Mental State Examination (MMSE),^[8]^ namely a normal cognitive function group (HNCF, n = 39, score 27 points or higher) and a cognitive dysfunction group (HCDF, n = 21, score < 27 points).^[9]^

The ethics approval was provided by the medical ethics committee of Qinghai Provincial People Hospital (2022-89) and written informed consent was acquired from all subjects.

#### 2.1.1. Inclusion criteria

Residents aged over 60 living in Maduo County, Qinghai Province, China at elevations of 4300 m. Subjects were required to pass the physical examination. The HAPC patients were required to be in line with the Qinghai diagnostic criteria for chronic plateau disease. Hemoglobin (Hb) levels for males are ≥210 g/L while those for females are 190 g/L.^[10,11]^ The healthy subjects were normal people with a Hb level of 150 to 190 g/L.^[11]^

#### 2.1.2. Exclusion criteria

Patients with acute or subacute altitude sickness. Patients with polycythemia vera or secondary polycythemia are caused by other diseases. Patients with malignant blood system diseases or patients with myeloproliferative diseases. Patients with Alzheimer disease, Parkinson disease, epilepsy, alcoholism, and other mental illnesses, as well as clinically asymptomatic lacunar cerebral infarction and leukoencephalopathy. Specimen hemolysis or precollection medication.

### 2.2. Research methods

#### 2.2.1. Data collection

Medical examination after general information is collected. The above-mentioned inclusion and exclusion criteria must be strictly followed based on the subjects’ detailed medical history.

#### 2.2.2. Determination of inflammatory cytokines

Blood samples were collected after physical examination, placed in clean and dry vacuum tubes without any additives, and kept stationary at room temperature for 1 hour. Serum was obtained after blood centrifugation at 3000 rpm for 10 minutes at 4°C. All samples were aliquoted and immediately stored at −80°C. After sample collection, the inflammatory factors were detected using ELISA kits from Dakewe Biotech Co., Ltd. (Shenzhen, China) according to the manufacturer’s protocols.^[12]^

#### 2.2.3. Cognitive test

The MMSE was used to assess cognitive function of the subjects. Each subject was required to answer 30 questions. A score of 1 was given for each correct answer, while a score of 0 was assigned for a wrong answer or no answer; thus, the maximum possible score was 30. Subjects with a score of 27 to 30 were considered to exhibit normal cognitive function, subjects with a score < 27 were considered to exhibit cognitive dysfunction, those with a score of 24 to 26 were considered to exhibit mild cognitive dysfunction, and those with a score < 24 were considered to exhibit severe cognitive dysfunction.^[9]^

### 2.3. Statistical treatment

SPSS18.0 was used for data analysis, and measurement data were expressed as mean ± standard deviation ($\bar{x}\pm s$). The *t* test was used to test for difference between the groups, the Chi-square test was used for counting the data, Pearson linear correlation analysis was used for correlation analysis, and *<*.01 was considered highly statistically significant and <.05 statistically significant.

## 3. Results

### 3.1. General information and MMSE scores

No statistically significant difference was observed between the 2 groups with respect to the general factors, such as gender, average age, and education level (all values of *P* > .05). In comparison with that in the control group, the MMSE scores of the subjects with HAPC were lower (*P* < .05), as presented in Table 1.

**Table 1:** Comparison of general information and MMSE scores between 2 groups ($\bar{x}\pm s$).

Group	n	Male/female	Age	Education level (yr)	MMSE scores
HAPC group	60	29/31	61.78 ± 0.57	5.88 ± 1.71	24.91 ± 2.66
Control group	60	30/30	60.19 ± 1.36	6.09 ± 1.39	27.15 ± 1.26
*t*	–	0.298	1.327	0.871	3.125
*P*	–	.981	.109	.612	.026

HAPC group: high-altitude polycythemia group, MMSE: mini-mental state examination.

### 3.2. Differential expression of inflammation factors among HAPC group and control group

**Figure 1.**
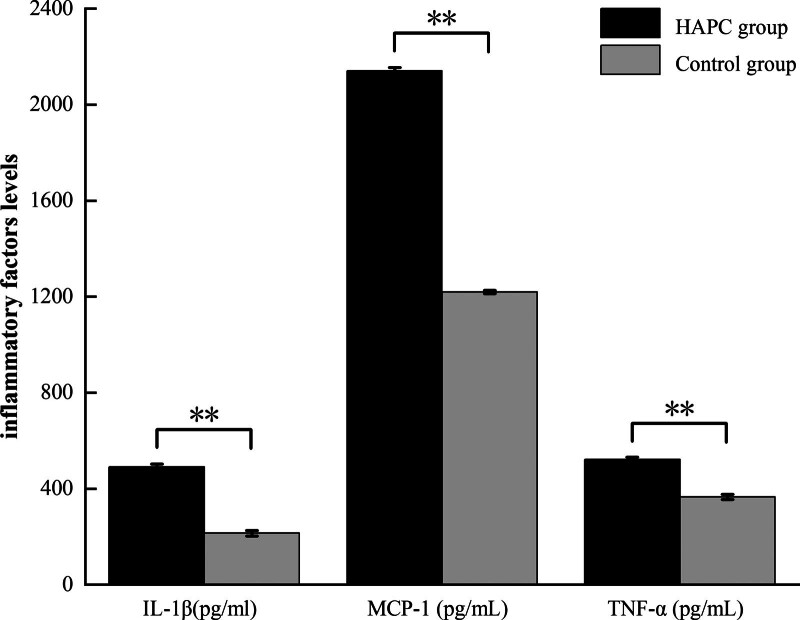
Comparison results of inflammatory factors between the 2 groups (IL-1β = interleukin-1beta, MCP-1 = monocyte chemoattractant protein-1, TNF-α = tumor necrosis factor alpha. HAPC group = high-altitude polycythemia group, n = 60; Control group, n = 60. [$\bar{x}\pm s$], **<.01).

The levels of interleukin-1beta (IL-1β), monocyte chemoattractant protein-1 (MCP-1), and tumor necrosis factor alpha (TNF-α) in the HAPC group were higher than those in the control group, and the differences between the 2 groups were statistically significant (Fig. 1, *P* < .01).

### 3.3. Comparison of inflammatory factors between the HNCF and HCDF

**Figure 2.**
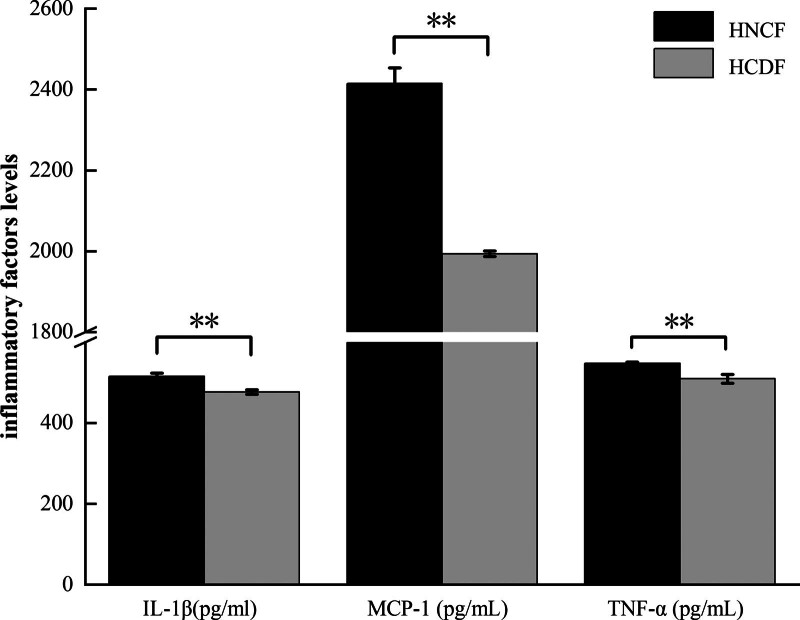
Comparison results of inflammatory factors between HNCF and HCDF (IL-1β = interleukin-1beta, MCP-1 = monocyte chemoattractant protein-1, TNF-α = tumor necrosis factor alpha. HNCF = normal cognitive function group, n = 21; HCDF = cognitive dysfunction group, n = 39. [$\bar{x}\pm s$], **<.01).

The levels of IL-1β, MCP-1, and TNF-α were higher in the HCDF in comparison with those in the HNCF, and the differences were statistically significant (Fig. 2, *P* < .01).

### 3.4. Analysis of correlation between inflammatory factors and MMSE scores

Pearson correlation analysis showed that the levels of IL-1β, MCP-1, and TNF-α were negatively correlated with the MMSE scores (Table 2, *P* < .05).

**Table 2:** Correlation analysis between inflammatory factors and MMSE scores.

Indicators	*r* value	*P* value
IL-1β	–0.092	.009
MCP-1	–0.875	.027
TNF-α	–0.190	.013

IL-1β: interleukin-1beta, MCP-1: monocyte chemoattractant protein-1, MMSE: mini-mental state examination, TNF-α: tumor necrosis factor alpha.

### 3.5. Analysis of correlation between inflammatory factors and Hb

The levels of IL-1β, MCP-1, and TNF- α were positively correlated with Hb (*P* < .05), as presented in Table 3.

**Table 3:** Correlation analysis results between inflammatory factors and Hb.

Indicators	*r* value	*P* value
IL-1β	0.921	.006
MCP-1	0.612	.007
TNF-α	0.447	.002

Hb: hemoglobin, IL-1β: interleukin-1beta, MCP-1: monocyte chemoattractant protein-1, TNF-α: tumor necrosis factor alpha.

## 4. Discussion

Existing studies have demonstrated that there are over 40 inflammatory factors that influence HAPC. Yu et al^[13]^ and Yi et al^[3]^ reported that the levels of IL-1β, IL-2, IL-3, IL-16, MCP-1, and TNF-α, were significantly up-regulated in HAPC patients. The up-regulated expression of inflammatory cytokines and the increased release of oxygen free radicals and reactive oxygen species lead to oxidative and enzymatic hydroxylation and other oxidative stress reactions,^[14]^ which eventually lead to the coexistence of oxygen-deficient injuries and inflammatory injuries. These reactions are particularly prominent in altitude hypoxia environments.

In this study, the results revealed that the levels of IL-1β, MCP-1, and TNF-α in the HAPC group were higher than those in the control group, and the differences were statistically significant (Fig. 1, *P* < .01). Furtherly, the level of IL-1β, MCP-1, and TNF-α were increased in the HAPC with HCDF than those in the HAPC with HNCF (Fig. 2, *P* < .01). Previous studies have demonstrated that IL-1 can induce the release of IL-2, stimulate the proliferation and differentiation of B cells, and promote the generation and release of inflammatory transmitters of various cells, thus causing inflammatory responses^[15,16]^ T cells are promoted by IL-2 to mature, proliferate, and induce immune tolerance.^[17]^ MCP-1 induces a specific chemotactic activation effect in mononuclear macrophages.^[18–20]^ TNF-α dominates the “TNF-α-TNFR1-TRADD-TRAF2-IP-TAK1IKK-NF-κB” signaling pathway, which mediates the pathophysiological process of inflammation.^[21,22]^ Therefore, we deduced that in a state of long-term hypoxia, IL-1 and IL-2, as the aggregation points of the inflammatory factor interaction network, play a pivotal role in the inflammatory response by factors, such as TNF- α, IL-15, IL-16, and MCP-1, which release a large amount of oxygen free radicals and Ros, causing imbalance of the internal environment of the body.^[23–25]^ Then, oxidative stress occurs, leading to the transformation of the body from oxygen-deficient injuries to coexistence with inflammatory lesions. Moreover, lipid peroxidation and enzyme hydroxylation of endothelial cells were induced, increasing capillary permeability, and thus exacerbating the progression of HAPC.

The altitude of Maduo County is over 4300 m. Residents residing on this plateau for a long time tend to “age early” and exhibit “senilism” due to severe environmental factors, such as hypoxia, low pressure, and ultraviolet radiation intensity. A significant amount of research attention has been attracted by the cognitive decline caused by anoxic environments. In this study, the responses of the patients with HAPC and healthy subjects to questions were evaluated using MMSE. In comparison with that in the control group, the MMSE scores of the HAPC group were lower (Table 1, *P* < .05), the Pearson correlation analysis showed that inflammatory factors were negatively correlated with MMSE (Table 2, *P* < .05), and positively correlated with Hb (Table 3, *P* < .01). Erythrocytosis is common in residents who have been residing in high-altitude hypoxia environments for a long time. Its secretion peripheral inflammation factors leads to inflammation in the brain, causing vascular endothelial damage and microvascular lesion and cerebral arteriosclerosis, cerebral perfusion, and cerebral damage metabolism. Long-term neuroinflammatory responses in the brain^[26]^ are an important pathogenic factor of neurodegenerative diseases. Studies have suggested that there exist a large number of inflammatory factors around Aβ plaques. Aβ and Tau can activate the glial cells to release inflammatory factors and inflammatory mediators to mediate synaptic damage and neuronal death; among them, IL-1, IL-6, and TNF-α are the major neuroinflammatory signaling proteins.^[27]^ Meanwhile, IL-1, IL-6, and TNF-α further activate the glial cells and promote the production of Aβ, forming a vicious circle in the pathological changes of dementia.^[28]^ Neuroinflammation is an important cause of accelerated brain degeneration and cognitive decline, which can lead to dementia, and controlling these inflammatory factors can delay cognitive decline.

## 5. Conclusion

Serum inflammatory factors were increased in HAPC, and inflammatory factors are positively correlated with Hb and negatively correlated with MMSE. We speculated that inflammatory factors are involved in the increase of Hb, which further leads to neurological impairment and cognitive dysfunction. Based on the experimental results, we can conclude that early detection and overall intervention of HAPC is a fundamental measure to reduce the increase in blood viscosity, microcirculation disorders, thrombosis, extensive organ damage, and cognitive dysfunction, among other disorders.

## Author contributions

**Data curation:** Yinglan Li, Jiabing Wang.

**Funding acquisition:** Jimei Li.

**Formal analysis:** Yinglan Li, Jiabing Wang.

**Investigation:** Yinglan Li, Jiabing Wang, Xiuxin Zhang, Qiong Ye, Yuan Yang, Xiaoshan Cui, Jinhua Feng, Jimei Li.

**Writing – original draft:** Yinglan Li, Jiabing Wang.

**Writing – review & editing:** Jimei Li.
